# Association of statin use and hypertriglyceridemia with diabetic macular edema in patients with type 2 diabetes and diabetic retinopathy

**DOI:** 10.1186/s12933-016-0486-2

**Published:** 2017-01-07

**Authors:** Yoo-Ri Chung, Sung Wook Park, Shin-Young Choi, Seung Woo Kim, Ka Young Moon, Jeong Hun Kim, Kihwang Lee

**Affiliations:** 1 Department of Ophthalmology, Ajou University School of Medicine, 164 World Cup-ro, Yeongtong-gu, Suwon, 16499 South Korea; 2 Fight Against Angiogenesis-Related Blindness (FARB) Laboratory, Clinical Research Institute, Seoul National University Hospital, Seoul, South Korea; 3 Department of Biomedical Sciences and Ophthalmology, Seoul National University College of Medicine, 101 Daehak-ro, Jongno-gu, Seoul, 03080 South Korea

**Keywords:** Diabetic macular edema, Diabetic retinopathy, Statin, Triglyceride

## Abstract

**Background:**

To investigate the effects of dyslipidemia and statin therapy on progression of diabetic retinopathy and diabetic macular edema in patients with type 2 diabetes.

**Methods:**

The medical records of 110 patients with type 2 diabetes (70 statin users and 40 non-users) were retrospectively reviewed. The two outcome measures were progression of diabetic retinopathy by two or more steps on the early treatment diabetic retinopathy study scale and diabetic macular edema based on optical coherence tomography. Serum lipid profiles were analyzed from 6 months prior to diagnosis of diabetic macular edema.

**Results:**

Diabetic retinopathy progressed in 23% of statin users and 18% of non-users (*p* = 0.506), but diabetic macular edema was present in 23% of statin users and 48% of non-users (*p* = 0.008). Statins reduced low-density lipoprotein cholesterol levels in patients with and without diabetic macular edema (*p* = 0.043 and *p* = 0.031, respectively). Among statin users, patients with diabetic macular edema had higher levels of triglycerides (*p* = 0.004) and lower levels of high-density lipoprotein cholesterol (*p* = 0.033) than those without diabetic macular edema. Logistic regression analysis showed that statin use significantly lowered the risk of diabetic macular edema [odds ratio (OR): 0.33, 95% confidence interval (CI) 0.12–0.91, *p* = 0.032]. Hypertriglyceridemia at 6 months prior to development of macular edema was significantly associated with central retinal thickness (OR: 1.52; 95% CI 1.14–2.02, *p* = 0.005).

**Conclusions:**

Lipid lowering therapy with statins protected against the development of diabetic macular edema and progression of diabetic retinopathy in patients with type 2 diabetes. Hypertriglyceridemia could be used as a surrogate marker for diabetic macular edema.

## Background

Lipid-lowering therapy with hydroxymethylglutaryl-CoA reductase inhibitors (statins) prevents major cardiovascular events and reduces mortality in patients with diabetes mellitus [1, 2]. However, recent studies found that statin use is associated with a small but significant increased risk for development of diabetes [3–5]. In contrast to these findings, the effect of statins on development of diabetic microvascular complications is unknown. A nationwide study in Denmark reported that statin users had a lower cumulative incidence of diabetic retinopathy (DR) and neuropathy before the diagnosis of diabetes [6]. On the other hand, Mansi et al. [5] reported that statin use was associated with an increased risk of diabetic complications in their study of propensity score-matched healthy statin users and non-users. Although these individual studies showed different results, the American College of Cardiology (ACC) and the American Heart Association (AHA) recently recommended statins for all people with diabetes between the ages of 40 and 75 years-old who have low-density lipoprotein (LDL) cholesterol of 70 mg/dL and above. If these guidelines are followed, it would make statins the second-most widely prescribed medication in the world, after hypoglycemic agents for treatment of diabetes [7]. However, the effect of statin use on the development of diabetic macular edema and progression of DR in patients with pre-existing type 2 diabetes is unknown.

DR is a microvascular complication of diabetes resulting from hyperglycemia and glucose-related hyperosmolarity, and diabetic macular edema is a leading cause of severe vision loss in patients with DR [8–10]. While the key factors for progression of DR and diabetic macular edema is known to be associated with the duration of diabetes and hypertension, dyslipidemia is considered a risk factor for DR and diabetic macular edema too [11–13]. Previous cohort studies suggested that increased levels of total cholesterol, triglycerides, and LDL cholesterol had positive associations with diabetic macular edema in patients with type 1 diabetes [14, 15]. The fenofibrate intervention and event lowering in diabetes (FIELD) study found that fenofibrate reduced the progression of DR in patients with type 2 diabetes [16, 17]. The action to control cardiovascular risk in diabetes (ACCORD) eye study examined the effects of fenofibrate on DR and showed that fenofibrate + simvastatin therapy slowed the progression of DR in patients with type 2 diabetes at 4 years [17, 18], although a follow-up study reported that fenofibrate provided no benefit at 8 years [17, 19]. A recent meta-analysis of the relationship of dyslipidemia with diabetic macular edema reported that most studies focused on the prevention of progression of DR, not on diabetic macular edema, because diabetic macular edema is not currently an indication for lipid lowering therapy [20].

The present study investigated the effect of statin administration and dyslipidemia on the development of diabetic macular edema in patients with type 2 diabetes. We also investigated the use of serum lipid profile as a surrogate maker for development of diabetic macular edema.

## Methods

The medical records of 1127 patients diagnosed with type 2 diabetes and followed-up by the Ophthalmology Department of Ajou University Hospital (Suwon, Korea) from January 2010 to October 2015 were retrospectively reviewed. This study was approved by the Institutional Review Board of Ajou University Hospital and complied with the Declaration of Helsinki. The following demographic and clinical factors were obtained from medical records: age, sex, duration of diabetes, follow-up period, presence of hypertension, treatment with hypoglycemic agents and/or statins, ocular treatment with intravitreal anti-vascular endothelial growth factor (VEGF) injections, HbA1c level, and serum lipid profile.

The severity of DR was graded using the early treatment DR study (ETDRS) scale, with seven-field stereo photographs and fluorescein angiography performed simultaneously [21]. Diabetic macular edema was defined as retinal thickening in the macular area of either eye, according to the ETDRS scale, and identified by optical coherence tomography (OCT) [22]. Patients with diabetic macular edema were defined as those who developed macular edema during the follow-up period. Central retinal thickness in cases with diabetic macular edema was measured as the distance from the hyperreflective line of the internal limiting membrane to the hyperreflective line of the retinal pigment epithelium/Bruch’s membrane complex using OCT [23]. Patients were excluded if they had follow-ups for less than 1 year, fewer than 2 fundus photographs, other retinal diseases, no DR, vitrectomy, use of lipid-lowering agents other than statins (such as fenofibrate, niacin, and/or fish oil), and no records on use of systemic medications.

The primary outcome measures were progression of DR by two or more steps on the ETDRS scale [16] and diabetic macular edema involving the fovea with central retinal thickness of 300 μm or more based on OCT findings. Serum lipid profiles and other laboratory data were analyzed from 6 months before to 12 months after diagnosis of diabetic macular edema to assess the effect of serum lipid profile. The condition of patients without diabetic macular edema was confirmed during entire study period, and laboratory data were analyzed at the time of the final fundus angiography.

Pearson correlation analysis and a generalized estimating equation model were used to analyze the correlation of variables with central retinal thickness in patients with diabetic macular edema at 1, 3 and 6 months prior to diagnosis. Logistic regression analysis was performed to evaluate the association between statin use and diabetic macular edema after controlling for age, duration of diabetes, HbA1c, and lipid profiles. Categorical variables were compared using the Chi square test, and continuous variables using the independent *t* test. Statistical analysis was performed using SPSS software (version 23.0, SPSS, Chicago, IL). Statistical significance was defined as a *p* value less than 0.05.

## Results

We initially examined the records 1127 patients with type 2 diabetes, and ultimately enrolled 110 patients (58 males and 52 females) who had DR. Table 1 summarizes demographic and clinical characteristics of patients who used statins (n = 70) and did not use statins (n = 40). Statin users were older (*p* = 0.016) and had a longer duration of diabetes (*p* = 0.019). DR progressed in 23% of statin users and 18% of non-users (*p* = 0.506). Notably, 16 of 70 statin users (23%) and 19 of 40 non-users (48%) showed diabetic macular edema based on OCT findings during the follow-up period (*p* = 0.008). Analysis of laboratory profiles indicated the statin users and non-users had no differences in the levels of HbA1c, triglycerides, and total and high-density lipoprotein (HDL) cholesterol. However, statin users had significantly lower levels of LDL cholesterol (*p* = 0.007).

**Table 1:** Baseline characteristics of patients with diabetic retinopathy (DR) who used or did not use statins

Variable	Statin group (n = 70)	No statin group (n = 40)	*p* value
Age	58.1 ± 11.6	52.3 ± 12.2	0.016^a^
Sex (male:female)	41:29	17:23	0.104
Duration of diabetes mellitus (years)	12.4 ± 8.0	8.6 ± 8.2	0.019^a^
Follow-up period (months)	26.8 ± 16.9	22.9 ± 15.4	0.251
Presence of hypertension	56/70 (80%)	26/40 (65%)	0.082
Initial DR severity scale			0.445
Mild NPDR (20–35)	17	5	
Mod-severe NPDR (43–53)	50	30	
PDR (more than 61)	3	5	
DR progression (≥2 steps of DRSS)	16/70 (23%)	7/40 (18%)	0.506
Presence of diabetic macular edema	16/70 (23%)	19/40 (48%)	0.008^a^
No. of IVT	1.2 ± 2.4	1.8 ± 3.0	0.317
HbA1c (%)	8.1 ± 1.7	8.0 ± 1.5	0.758
Total cholesterol (mmol/L)	4.4 ± 2.6	4.4 ± 1.5	0.904
Triglycerides (mmol/L)	1.9 ± 1.4	2.1 ± 1.5	0.569
HDL cholesterol (mmol/L)	1.1 ± 0.3	1.1 ± 0.3	0.990
LDL cholesterol (mmol/L)	2.1 ± 0.8	3.0 ± 1.3	0.007^a^

*DR* diabetic retinopathy, *DRSS* diabetic retinopathy severity scale, *HDL* high-density lipoprotein, *IVT* intravitreal injection of anti-VEGF agents, *LDL* low-density lipoprotein, *NPDR* non-proliferative diabetic retinopathy, *PDR* proliferative diabetic retinopathy

^a^ *p* value <0.05 (independent *t*-test or Chi square test)

Subgroup analysis of patients with and without diabetic macular edema indicated that LDL cholesterol was significantly lower in statin users with and without diabetic macular edema (*p* = 0.043 and *p* = 0.031, respectively, Table 2). These results suggest that lipid lowering therapy with statins protected against the development of diabetic macular edema without increasing the risk of DR progression.

**Table 2:** Serum lipid profiles of patients with diabetic retinopathy who had or did not have diabetic macular edema (DME) and who used or did not use statins

Variable	Statin	No statin	*p* value
DME group (no. of patients)	16	19	
HbA1c (%)	7.7 ± 1.2	7.9 ± 1.6	0.642
Total cholesterol (mmol/l)	4.7 ± 1.5	5.0 ± 1.2	0.501
Triglycerides (mmol/l)	3.0 ± 2.7	2.3 ± 1.4	0.388
HDL cholesterol (mmol/l)	0.9 ± 0.3	1.0 ± 0.4	0.410
LDL cholesterol (mmol/l)	2.2 ± 0.8	3.2 ± 1.1	0.043^a^
No DME group (no. of patients)	54	21	
HbA1c (%)	8.2 ± 1.7	8.2 ± 1.7	0.932
Total cholesterol (mmol/L)	4.3 ± 2.8	4.2 ± 1.6	0.866
Triglycerides (mmol/L)	1.7 ± 0.9	1.9 ± 0.8	0.246
HDL cholesterol (mmol/L)	1.1 ± 0.4	1.2 ± 0.3	0.916
LDL cholesterol (mmol/L)	2.1 ± 0.8	2.8 ± 1.3	0.031^a^

*DME* diabetic macular edema, *HDL* high-density lipoprotein, *LDL* low-density lipoprotein

^a^ *p* value <0.05 (independent *t*-test)

Despite this beneficial effect of statins, analysis of statin users indicated that those with diabetic macular edema had a significantly higher level of triglycerides (*p* = 0.004) and a lower level of HDL cholesterol (*p* = 0.033) (Table 3). This result suggests that hypertriglyceridemia, rather than dyslipidemia, may lead to the development of diabetic macular edema in statin users. There were similar trends in non-users of statins, but the differences were not statistically significant (data not shown). Logistic regression analysis was performed to evaluate factors associated with diabetic macular edema (Table 4). Statin use was associated with a significantly lower risk of diabetic macular edema (odd ratio (OR): 0.40; 95% confidence interval (CI) 0.17–0.90; *p* = 0.028). Triglycerides level was associated with diabetic macular edema, while HDL cholesterol level lowered the risk. When adjusted with age, duration of diabetes, HbA1c, triglycerides and HDL cholesterol in multivariate analysis, statin use had a significant protective effect on diabetic macular edema (OR: 0.33; 95% CI 0.12–0.91; *p* = 0.032).

**Table 3:** Serum lipid profiles of patients with diabetic retinopathy who used or did not use statins and who had or did not have diabetic macular edema (DME)

Variable	DME	No DME	*p* value
Statin group (no. of patients)	16	54	
HbA1c (%)	7.7 ± 1.2	8.2 ± 1.7	0.282
Total cholesterol (mmol/L)	4.7 ± 1.5	4.3 ± 2.8	0.603
Triglycerides (mmol/L)	3.0 ± 2.7	1.7 ± 0.9	0.004^a^
HDL cholesterol (mmol/L)	0.9 ± 0.3	1.1 ± 0.4	0.033^a^
LDL cholesterol (mmol/L)	2.2 ± 0.8	2.1 ± 0.8	0.761
No-statin group (no. of patients)	19	21	
HbA1c (%)	7.9 ± 1.6	8.2 ± 1.7	0.649
Total cholesterol (mmol/L)	5.0 ± 1.2	4.2 ± 1.6	0.092
Triglycerides (mmol/L)	2.3 ± 1.4	1.9 ± 0.8	0.349
HDL cholesterol (mmol/L)	1.0 ± 0.4	1.2 ± 0.3	0.219
LDL cholesterol (mmol/L)	3.2 ± 1.1	2.8 ± 1.3	0.443

*DME* diabetic macular edema, *HDL* high-density lipoprotein, *LDL* low-density lipoprotein

^a^ *p* value <0.05 (independent *t*-test)

**Table 4:** Logistic regression analysis between patients with and without diabetic macular edema for variables associated with diabetic macular edema

Variable	OR (95% CI)	*p* value
Age	1.00 (0.97–1.04)	0.892
Duration of diabetes	1.00 (0.95–1.05)	0.843
Statin use	0.40 (0.17–0.90)	0.028^a^
HbA1c	0.98 (0.95–1.00)	0.091
Total cholesterol	1.11 (0.92–1.35)	0.289
Triglycerides	1.51 (1.06–2.14)	0.023^a^
HDL cholesterol	0.18 (0.04–0.80)	0.024^a^
LDL cholesterol	1.48 (0.89–2.44)	0.132

*HDL* high-density lipoprotein, *LDL* low-density lipoprotein

^a^ *p* value <0.05 (logistic regression analysis)

We also examined serum lipid profiles and HbA1c levels from 6 months prior to 12 months after diagnosis of diabetic macular edema in all patients to investigate the effect of these variables on disease progression (Table 5). The results indicate that the levels of HbA1c, triglycerides, total cholesterol, and HDL cholesterol correlated with central retinal thickness. Specifically, the levels of triglycerides at 6 months prior to diabetic macular edema, HbA1c at the onset and 3 months prior to diabetic macular edema, and total cholesterol at the onset and 1 month prior to diabetic macular correlated positively with central retinal thickness; the HDL cholesterol level at 3 months prior to diabetic macular edema had a negative correlation with central retinal thickness. Furthermore, analysis using a generalized estimating equation indicated that only hypertriglyceridemia at 6 months prior to development of macular edema was associated with central retinal thickness (OR 1.52; 95% CI 1.14–2.02, *p* = 0.005). Taken together, these results indicate that hypertriglyceridemia is associated with the incidence and severity of diabetic macular edema in patients with type 2 diabetes who are taking statins.

**Table 5:** Correlation of serum variables with central retinal thickness in patients with diabetic macular edema

Variable	Months	R	*p* value
HbA1c	−3 M, 0 M	0.709	0.022^a^
Total cholesterol	−1 M, 0 M	0.516	0.028^a^
Triglycerides	−6 M	0.559	0.010^a^
HDL cholesterol	−3 M	−0.715	0.009^a^
LDL cholesterol		0.150	0.700

*HDL* high-density lipoprotein, *LDL* low-density lipoprotein. The time of diagnosis with diabetic macular edema is ‘0 M’, 1, 3, and 6 months prior to diabetic macular edema are presented as ‘−1 M’, ‘−3 M’, and ‘−6 M’

^a^ *p* value <0.05 (Pearson correlation analysis)

## Discussion

Dyslipidemia is common in patients with type 2 diabetes and is a well-known risk factor for atherosclerosis and cardiovascular diseases [24, 25]. Treatments that lower cholesterol, especially LDL cholesterol, reduce the risk of cardiovascular morbidity in many patient populations, although there are controversies on the overall benefits of intensive statin therapy [25, 26]. However, statin use seems to modestly increase the risk of new-onset type 2 diabetes in individuals with a pre-existing elevated risk for diabetes, probably because they increase insulin resistance and decrease insulin secretion [3, 4]. However, the benefits of reduced risk of cardiovascular disease probably outweigh the potentially increased risk of diabetes [1]. A recent large retrospective cohort study with a follow-up period of 6.5 years reported that statin use significantly increased the risk of new-onset diabetes and diabetic complications in healthy adults [5]. Thus, statin use may be a serious concern for individuals with predispositions for diabetes or pre-existing diabetes, but few studies have examined the effect of statins on diabetic microvascular complications, especially DR and diabetic macular edema.

The present study of patients with type 2 diabetes and DR shows that statin users have lower levels of LDL cholesterol than non-users. Notably, our results also show that statin therapy reduces the prevalence of diabetic macular edema in patients with type 2 diabetes and pre-existing DR, but does not contribute to the progression of DR. Considering that our statin users had a significantly longer duration of diabetes than non-users (Table 1), and that duration of diabetes is the strongest predictor for development and progression of DR [27], these results suggest that statins protect against the development of diabetic macular edema and progression of DR. Statins may protect against microvascular damage in patients with diabetes for several reasons. First, statins may benefit dyslipidemic patients with diabetes because they may reduce lipid leakage and improve lipid clearance in the eyes [28, 29]. Second, statins increase HbA1c in about 0.3% in patients with diabetes, so that may only cause relatively small exacerbations of diabetes [2]. Lastly, statins have anti-inflammatory effects, and this might slow the progression of microvascular complications in patients with diabetes [30, 31].

Previous studies showed that dyslipidemia is associated with diabetic macular edema [14, 32]. In addition, a global multicenter study reported that a high level of triglycerides and a low level of HDL cholesterol were associated with increased risk of diabetic microvascular diseases [33]. In our population, it is important to note that statin users with diabetic macular edema had a significantly higher level of triglycerides (*p* = 0.004) and a lower level of HDL cholesterol (*p* = 0.033) than those without diabetic macular edema, even though LDL cholesterol levels were well-controlled in both groups. A recent meta-analysis of cohort and case–control studies in patients with type 1 and 2 diabetes showed that serum triglyceride level was an independent risk factor for diabetic macular edema [20]. Moreover, a study of patients with type 1 diabetes reported no evidence of a relationship between oxidized LDL level and the incidence of diabetic macular edema or worsening of DR [34]. These findings motivated us to analyze the possible use of serum triglyceride level as a surrogate marker for macular edema in patients with type 2 diabetes. Indeed, serum triglycerides level at 6 months prior to the diagnosis of diabetic macular edema had a positive correlation with development of diabetic macular edema and with increased central retinal thickness in patients with diabetic macular edema. These results suggest that serum triglyceride level could be used as a surrogate marker for the development and severity of macular edema.

Our findings show that triglycerides and LDL cholesterol play important roles in the onset and severity of diabetic macular edema. Lipid lowering therapy with statins targets LDL cholesterol [7, 24, 25], but our findings indicate that the triglyceride level must also be controlled to prevent diabetic macular edema in patients with DR. Current treatment guidelines for dyslipidemia focus on LDL cholesterol and use of statin therapy [24, 25]. New guidelines released by the ACC/AHA in November 2013 recommend statin therapy for all patients with diabetes who are 40–75 years-old, have LDL cholesterol levels of 70 mg/dL or more, and have a 10-year risk of cardiovascular disease risk that is 7.5% or more, based on pooled cohort equations [7, 25]. These guidelines also suggest use of other therapies for patients with type 2 diabetes, such as fibrates, niacin, and/or fish oil, when the serum triglyceride level is moderately high despite statin use. Statin therapy can reduce triglycerides level by 10–20%, depending on the specific statins [35–39]. More specifically, rosuvastatin, atorvastatin, and especially pitavastatin are more effective in reducing triglyceride levels than the older statins [35–42]. The greatest benefit was seen in patients with high baseline triglyceride levels [35–42]. Furthermore, the combined use of statin and a fibrate (e.g. fenofibrate and bezafibrate) more effectively lowers serum triglycerides than statin therapy alone [43–45]. The ACCORD trial reported that fenofibrate provided no overall benefit for patients with type 2 diabetes when added to a statin, but it did improve outcomes in a subset of patients with elevated triglycerides (>204 mg/dL [2.30 mmol/L]) and low HDL cholesterol levels (<34 mg/dL [0.88 mmol/L]) [17, 46]. Based on these considerations, we suggest use of a statin with a supplemental therapy for patients with hypertriglyceridemia and DR to protect against diabetic macular edema.

Targeting triglycerides as a therapeutic indication for patients with type 2 diabetes would also help to reduce macrovascular complications such as cardiovascular events. Many epidemiological studies showed that DR is associated with macrovascular diseases in patients with type 2 diabetes [47]. Patients with type 2 diabetes and DR have an increased presence and number of plaques in the carotid territory [48]. The presence of dyslipidemia (elevated triglycerides and decreased HDL cholesterol) is associated with silent myocardial ischemia or angiographic coronary artery disease in asymptomatic patients with type 2 diabetes [49].

This study has several limitations. The small number of patients with diabetic macular edema is the major limitation. Thus, it might be difficult to generalize our results to other populations of patients with diabetic macular edema. However, our results showed significant relationships between serum triglycerides and diabetic macular edema, in agreement with the findings of the FIELD study and a recent meta-analysis of risk factors for diabetic macular edema [16, 20]. The present study also had a retrospective design, and the patients used different doses and different kinds of statins. These limitations could be overcome by performing a prospective randomized controlled trial. In addition, large and long-term randomized controlled prospective studies are needed to obtain a more complete risk/benefit assessment of the effect of statin therapy on DR and diabetic macular edema. Several previous trials have compared standard-dose versus high-dose statin therapy for prevention of cardiovascular diseases [50, 51]. Similar studies are needed for DR and diabetic macular edema.

## Conclusions

In conclusion, use of statins by patients with type 2 diabetes and pre-existing DR did not increase the risk of DR progression, but was protective against development of diabetic macular edema. Hypertriglyceridemia is a potential surrogate marker for diabetic macular edema in patients with type 2 diabetes. Moreover, clinicians should closely monitor hypertriglyceridemia in patients with type 2 diabetes and taking statins. Use of a statin with a supplemental therapy (fenofibrate, niacin, and/or fish oil) should be considered for patients with DR to prevent the onset and reduce the severity of diabetic macular edema.

## Abbreviations

ACC: American College of Cardiology
ACCORD: action to control cardiovascular risk in diabetes
AHA: American Heart Association
CI: confidence interval
DR: diabetic retinopathy
ETDRS: early treatment diabetic retinopathy study
FIELD: fenofibrate intervention and event lowering in diabetes
HDL: high-density lipoprotein
LDL: low-density lipoprotein
OCT: optical coherence tomography
OR: odd ratio
VEGF: vascular endothelial growth factor
